# Editorial: Insights in neuropsychology 2021

**DOI:** 10.3389/fpsyg.2023.1225520

**Published:** 2023-06-28

**Authors:** Sara Palermo, Sara Bottiroli, Lawrence M. Parsons, Matthew Justin Wright, Martina Amanzio

**Affiliations:** ^1^Department of Psychology, University of Turin, Turin, Italy; ^2^Neuroradiology Unit, Department of Diagnostic and Technology, Fondazione IRCCS Istituto Neurologico Carlo Besta, Milan, Italy; ^3^Faculty of Law, Giustino Fortunato University, Benevento, Italy; ^4^Headache Science and Neurorehabilitation Center, IRCCS Mondino Foundation, Pavia, Italy; ^5^Department of Psychology, The University of Sheffield, Sheffield, United Kingdom; ^6^Harbor–UCLA Medical Center, Torrance, CA, United States; ^7^Lundquist Institute for Biomedical Innovation, Torrance, CA, United States

**Keywords:** digital assessment, visual memory cognitive task, dual-tasking, fluency, neurocognitive abnormalities, theory of mind (ToM), tuberous sclerosis complex

In recent years, achievements in the fields of neuropsychology have been rapid and exceptional, leading to major advances in the clinical-application field. This editorial initiative is focused on new insights, novel developments, current challenges, latest discoveries, recent advances and future perspectives in the field of neuropsychology. The Research Topic was intended to solicit contributions that describe the state of the art, outlining recent developments and suggesting what needs to be achieved to advance the field.

The goal of this special edition Research Topic is to shed light on the progress made in the past decade in the Neuropsychology field and on its future challenges to provide a thorough overview of the state of the art in this area of research. This article collection will inspire, inform, and provide direction and guidance to researchers in the field. This collection comprises eight contributions consisting of three original research articles, three reviews, one brief research report, and one perspective. Overall, the scientific contributions published in this Research Topic exemplify the great complexity hidden behind the challenges that neuropsychology is facing.

An initial review offers a critical and historical review of existing measures and resources for neuropsychological assessment of visual/visuo-spatial memory and presents examples of more recent tests that have attempted to overcome the challenges of assessing these important aspects of memory (Diaz-Orueta et al.). The authors also identify new trends and examples of how technological advances, such as virtual reality, can add value in overcoming previous barriers to assessment, thus offering professionals more reliable and accurate means of assessing visual/visuo-spatial memory in clinical practice (Diaz-Orueta et al.). Indeed, computerized neuropsychological tests on digital platforms are sensitive and cost-effective and could be of great use in cohort studies and clinical trials (e.g., Bottiroli et al., 2017, 2021). The aim of a first original research was to compare sensitivity in detecting contralesionally omissions of two different computer-based methods: a “digitally converted” cancellation task was compared with a computer-based Visual and Auditory dual-tasking approach, which has already proved to be very sensitive (Villarreal et al.). Traditionally, asymmetric spatial processing (i.e., hemispatial neglect) has been assessed with paper-and-pencil tasks, but growing evidence indicates that computer-based methods are a more sensitive mode of assessment. Authors concluded that Attentionally demanding methods are useful for revealing mild forms of contralesional visuospatial deficits (Villarreal et al.).

Increased attention to the manner of test administration, scoring, and interpretation is emphasized, especially in the case of specific sociocultural settings and needs. the case that arises, for example, when neuropsychologists have to assess reading ability in patients with tuberous sclerosis complex in a non-Western culture patient (Lee et al.). The study applied the Chinese character fluency test to measure children's word recognition and reading comprehension to observe whether they exhibit the characteristics of reading disability, as an indicator of the spectrum of reading ability in patients (Lee et al.). Also, in childhood is the third proposed research (Starowicz-Filip et al.). The authors suggested the possible presence of emotional-behavioral alterations in children with cerebellar damage, which are the most similar to some behaviors observed in autism spectrum disorders. They showed that the executive functions most sensitive to cerebellar lesions are divided attention, planning ability, and impulsivity control (Starowicz-Filip et al.).

The first of the published systematic reviews was aimed at abstracting and evaluating models for predicting radiation-induced neurocognitive decline in patients with primary or secondary brain tumors (Tohidinezhad et al.). The authors identified as many as 23 prediction models to estimate the risk of neurocognitive decline after radiation therapy in patients with primary or secondary brain tumors. The models have substantial heterogeneity in terms of outcome assessment and were found to have a relatively high risk of bias (Tohidinezhad et al.). The second systematic review aimed to analyze the functionality of theory of mind in patients with mild cognitive impairment (Morellini et al.). The review also aimed to group the variety of task types used by the author to assess multiple domains of the Theory of Mind.

The absence of objective cognitive impairment distinguishes Subjective Cognitive Decline (SCD) from mild cognitive impairment (Jessen et al., 2020). SCD can be considered as an independent risk factor for mild cognitive impairment (Li et al., 2022). In a brief research report article, Authors proposed a new screening tool for SCD (Maffoni et al.). Little is known about change in prospective memory, following reports of SCD (Hsu et al., 2015; Kamberis et al., 2021). In a perspective article, the authors discussed the prospective memory paradox, where older adults outperformed younger people on some tasks, but not others (Blondelle et al.). These data are of utmost importance if we think in terms of post-pandemic effects (Amanzio et al., 2021; Palermo et al., 2023). In particular, loneliness leaves a “signature” in terms of changes in both the volume and type of connections of specific brain areas (Spreng et al., 2020; Morese and Palermo, 2022) and negatively impact on metacognitive-executive functions (Amanzio et al., 2017; Morese et al., 2018; Palermo et al., 2018; Palermo, 2022). Since the beginning of the pandemic, the intervention of psychologists in managing and containing the infection sequalae has been essential. SARS-CoV-2 affects the central nervous system and cognitive, emotional, and behavioral functions could be severely impaired. Understanding these issues and learning how to deal with them is the challenge ahead.

## Author contributions

All authors listed have made a substantial, direct, and intellectual contribution to the work and approved it for publication.
